# Human non-small cell lung cancer expresses putative cancer stem cell markers and exhibits the transcriptomic profile of multipotent cells

**DOI:** 10.1186/s12885-015-1086-3

**Published:** 2015-02-25

**Authors:** Norashikin Zakaria, Narazah Mohd Yusoff, Zubaidah Zakaria, Moon Nian Lim, Puteri J Noor Baharuddin, Kamal Shaik Fakiruddin, Badrul Yahaya

**Affiliations:** 1 Regenerative Medicine Cluster, Advanced Medical and Dental Institute (AMDI), Universiti Sains Malaysia, Bertam, 13200 Kepala Batas, Pulau Pinang Malaysia; 2 Stem Cell Laboratory, Haematology Unit, Cancer Research Centre, Institute for Medical Research (IMR), Kuala Lumpur, Malaysia

**Keywords:** Cancer stem cells, Non-small cell lung cancer, Cell surface marker, Transcriptome

## Abstract

**Background:**

Despite significant advances in staging and therapies, lung cancer remains a major cause of cancer-related lethality due to its high incidence and recurrence. Clearly, a novel approach is required to develop new therapies to treat this devastating disease. Recent evidence indicates that tumours contain a small population of cells known as cancer stem cells (CSCs) that are responsible for tumour maintenance, spreading and resistant to chemotherapy. The genetic composition of CSCs so far is not fully understood, but manipulation of the specific genes that maintain their integrity would be beneficial for developing strategies to combat cancer. Therefore, the goal of this study isto identify the transcriptomic composition and biological functions of CSCs from non-small cell lung cancer (NSCLC).

**Methods:**

We isolated putative lung CSCs from lung adenocarcinoma cells (A549 and H2170) and normal stem cells from normal bronchial epithelial cells (PHBEC) on the basis of positive expression of stem cell surface markers (CD166, CD44, and EpCAM) using fluorescence-activated cell sorting. The isolated cells were then characterised for their self-renewal characteristics, differentiation capabilities, expression of stem cell transcription factor and *in vivo* tumouregenicity. The transcriptomic profiles of putative lung CSCs then were obtained using microarray analysis. Significantly regulated genes (p < 0.05, fold change (FC) > 2.0) in putative CSCs were identified and further analysed for their biological functions using the Database for Annotation, Visualization, and Integrated Discovery (DAVID).

**Results:**

The putative lung CSCs phenotypes of CD166^+^/CD44^+^ and CD166^+^/EpCAM^+^ showed multipotent characteristics of stem cells, including the ability to differentiate into adipogenic and osteogenic cells, self-renewal, and expression of stem cell transcription factors such as Sox2 and Oct3/4. Moreover, the cells also shows the *in vivo* tumouregenicity characteristic when transplanted into nude mice. Microarray and bioinformatics data analyses revealed that the putative lung CSCs have molecular signatures of both normal and cancer stem cells and that the most prominent biological functions are associated with angiogenesis, migration, pro-apoptosis and anti-apoptosis, osteoblast differentiation, mesenchymal cell differentiation, and mesenchyme development. Additionally, self-renewal pathways such as the Wnt and hedgehog signalling pathways, cancer pathways, and extracellular matrix (ECM)-receptor interaction pathways are significantly associated with the putative lung CSCs.

**Conclusion:**

This study revealed that isolated lung CSCs exhibit the characteristics of multipotent stem cells and that their genetic composition might be valuable for future gene and stem cells therapy for lung cancer.

**Electronic supplementary material:**

The online version of this article (doi:10.1186/s12885-015-1086-3) contains supplementary material, which is available to authorized users.

## Background

Lung cancer is one of the most common malignancies throughout the world. It accounted for about 16.1 million deaths in 2008 and is the leading cause of cancer-related death [1]. Based on pathological features, lung cancer is classified into two major groups; small cell lung carcinoma (SCLC) and non-small cell lung carcinoma (NSCLC). The majority of lung cancer cases are NSCLC (80%). NSCLC is less aggressive than SCLC. NSCLC tends to grow and spread slower than SCLC, which is fast growing and rapidly spreads to the bloodstream and other parts of the body. The three main subtypes of NSCLC are adenocarcinoma, squamous cell carcinoma, and large-cell carcinoma. The prognosis for patients with NSCLC remains very poor, with only 15% survival within 5 years after treatment [2,3]. Moreover, the recurrence rate ranges from 35% to 50% among early stage NSCLC patients: After an apparently successful initial therapy, development of secondary tumours often leads to a lethal relapse.

The biological characteristics associated with the aggressive behaviour of cancer cells is driven by a subpopulation of cells within the tumour called cancer stem cells (CSCs) [4,5]. CSCs were first described in human hematopoietic cancer, and to date they have been identified in solid tumours of breast [6], pancreas [7], brain [8], and colon [9,10] cancers. CSCs can self-renew, initiate tumour development, and differentiate into multiple cell types [4,11-13], and recent evidence suggests that these cells play a central role in the progression of malignant tumours. The CSCs model describes the existence of a small subpopulation of plastic cells with transdifferentiation potential in tumours. However, recent studies suggest that a major proportion of cells within tumours maintain stem cell properties and even more differentiated cells can be transformed into stem-like cells [13,14]. If this is the case, eradication of CSCs might not be a useful strategy for the reduction of tumour growth. Therefore, it is important to understand CSCs biology and identify new strategies to prevent malignant tumour progression. The mechanisms that regulate self-renewal of both CSCs and normal stem cells are thought to be similar [4].

Currently, identification and isolation of CSCs is largely dependent on the presence of specific cell surface markers [10,15], although the expression of such markers depends on various factors (e.g., the differentiation state of the cells and niche factors). Many of the markers used to identify CSCs are derived from the surface markers known to be present on normal hematopoietic or embryonic stem cells. CD133 has been used as a putative stem cell marker in glioblastoma [16] and colon cancer [9]; CD34 expressing tumour epithelial cells have been used as a marker in cutaneous cancer [17]; and CD44 expressing cells have been used as a marker in breast cancer [18]. Moreover, CD26 positive cells are indicative of metastases, invasiveness, and chemoresistance in colon cancer, and CD271 positive cells initiate melanoma progression and metastasis [19]. For lung CSCs, CD133 [20], CD166 [21], EpCAM, CD90, and CD44 [15,22] have been used as markers. CD133 is a well-described CSCs marker in various types of cancers, including hematopoietic [23], brain [11], colon [10], pancreatic [7], and lung [20] cancers. In NSCLC and SCLC patient samples, CD133^+^ cells possess tumourigenic and self-renewal characteristics [20]. However, several studies suggest that the use of CD133 expression to discriminate lung CSCs is overstated. For example, some CD133^−^ lung cancer cells also possess the ability to self-renew and generate the formation of xenograft when transplanted into recipient mice [24]. Unlike in gliomas, where CD133 is a more established cancer stem cell marker, CD133 expression in lung cancer is not associated with patient prognosis [25-27]. Moreover, in many lung cancer samples, CD133 is not detected [26-28]. Recently, few scientists have questioned the use of CD133 as a selective CSCs marker in other solid tumour types, citing cases where CD133ˉ cells also possess the capacity for self-renewal and cancer initiation [29,30]. Based on these data, we exclude CD133 in this study and focus only on CD166, CD44, and EpCAM.

The goal of this study is to identify and characterise the CSCs population in human NSCLC using CD166, CD44, and EpCAM as markers. We also conducted transcriptomic profiling of the isolated CSCs to determine how the transcriptome is involved in the signaling pathways specific to the CSCs of lung cancer.

## Methods

### Cell lines

The human lung cancer cell lines A549 (lung carcinoma) and H2170 (squamous cell carcinoma) and the normal primary human bronchial/tracheal epithelium (PHBEC) cell line were purchased from the American Type Culture Collection (ATCC, Manassas, VA, USA).

### Cell culture

The cancer cell lines were cultured in RPMI-1640 medium supplemented with 10% fetal bovine serum (FBS) and 1% penicillin/streptomycin. Cells were incubated in a humidified incubator at 37°C and 5% CO_2_. Cells were maintained in 75 cm^2^ tissue culture flasks and harvested by 0.25% trypsin-EDTA treatment when they reached 80% confluency. Unless specified, all reagents were obtained from Gibco (Life Technologies, Foster City, CA, USA).

PHBECs were cultured in specific airway epithelial cell medium purchased from ATCC. The medium consists of airway epithelial cell basal medium (PCS-300-030) supplemented with the bronchial/tracheal epithelial growth kit (PCS-300-040), gentamicin-amphoterin B solution (PCS-999-025), penicillin-streptomycin-amphoterin B Solution (PCS-999-002), and phenol red (PCS-999-001). The cells were incubated in a humidified incubator at 37°C and 5% CO_2_. Cells were maintained in 75 cm^2^ tissue culture flasks and harvested using 0.05% Trypsin-EDTA when they reached 80% confluence.

### Isolation of putative CSCs and normal stem cells

The lung cancer cells and normal cells were detached with trypsin and washed with phosphate buffer solution (PBS) containing 2% FBS (PBS/2% FBS). The cell suspensions were then labelled with antibodies CD44-FITC (Clone: L178; Isotype: Mouse IgG1, κ), CD166-PE (Clone: 3A4; Isotype: Mouse IgG1, κ) (BD Biosciences, San Jose, CA, USA), and EpCAM–FITC (Clone: 158206; Isotype: Mouse IgG_2B_; Isotype: Mouse IgG1, κ) (R&D System, Minneapolis, MN, USA). Briefly, the cells were resuspended in 90 μL of PBS/2% FBS. Next, 10 μL of each antibody were added to the cell suspensions and incubated for 30 min on ice and in the dark. At the end of the incubation, unbound antibodies were washed away with PBS. Each cells pellet was resuspended in 300–500 μL PBS/2% FBS and filtered through a 40 μm cell strainer to obtain a single cell suspensions before sorting. The expression of cancer stem cell markers (CD166, CD44, and EpCAM) was analysed and populations of cells expressing the markers were sorted using a fluorescence-activated cell sorter (FACSAria III, BD Biosciences). The sorting for each cell population was done in three independent experiments to represent the biological variation.

### Adipogenic, chondrogenic, and osteogenic differentiation *in vitro*

The putative CSCs were induced to differentiate into different lineages using adipogenic, chondrogenic, and osteogenic differentiation media (PromoCell, Heidelberg Germany). Briefly, the putative CSCs were seeded in 24-well tissue culture plates until the cells reached 80–90% confluence (for adipogenic differentiation) or 100% confluence (for chondrogenic and osteogenic differentiation). The initial seeding number was 6 × 10^4^ cells for adipogenic and chondrogenic differentiation and 1 × 10^5^ cells for osteogenic differentiation. Once the cells reached the required confluency, two sets of triplicate wells were induced to differentiate by replacing the culture medium with the specific differentiation medium. The remaining wells containing the normal medium served as the control. The cells were incubated for 14 days (adipogenic differentiation) and 21 days (chondrogenic and osteogenic differentiation), and the medium was changed every 3 days.

### Detection of differentiation *in vitro*

At the end of the incubation period, the cells were washed with PBS, fixed with 10% buffered formalin, and stained with a respective staining solution to detect adipocyte, chondrocyte, or osteocyte formation. Formation of adipocytes was detected by observing intracellular lipid vesicles stained red by 0.3% Oil Red O (Sigma-Aldrich, Munich, Germany). After the cells were fixed, they were incubated with 60% isopropanol at room temperature for 5 min. The isopropanol was carefully aspirated, and Oil Red O staining solution was added to cover the cells. The cells were incubated for 15 min, washed several times with distilled water, and counterstained with a Harris Hematoxylin solution for 1 min. Lastly, the cells were washed with distilled water and observed under the microscope.

Osteocyte formation was detected by staining calcium deposits with 2% Alizarin Red S (Sigma-Aldrich). The fixed cells were incubated with Alizarin Red S staining solution for 45 min at room temperature in the dark. The cells were washed four times with distilled water, and PBS was then added to each well. When observed under the microscope, extracellular calcium deposits were stained bright orange-red. Chondrogenic differentiation was detected by staining with Alcian blue staining solution (Sigma-Aldrich). The fixed cells were incubated with Alcian blue staining solution overnight at room temperature in the dark. The cells were washed four times with distilled water, and PBS then was added to each well. When observed under the microscope, the cartilages were stained an intense dark-blue, whereas other tissue was at most faintly bluish.

### Colony forming assay

For the colony forming assay, the cells were trypisinised as described previously. The cells were seeded in 6-well plates at low density (~200 cells per well) and cultured for 7 days. The plates were then washed with PBS and fixed with 10% formalin for 10 min followed by staining with crystal violet for 30 min. The plates were then washed with PBS, and images of each well were captured using an inverted microscope. The experiment was performed in three independent replicates for A549 and H2170 cells.

### Sphere forming assay

Isolated putative lung CSCs were cultured in low adherent 35 mm dishes under serum-free conditions and supplemented with 20 ng/ml of epidermal growth factor (EGF) (Life Technologies, Foster City, CA, USA) 10 ng/ml of basic fibroblast growth factor (bFGF) (Life Technologies), and B27 supplement (Life Technologies) for 21 days according to published protocols [15]. The experiment was conducted in three independent replicates for A549 and H2170 cells.

## Expression of the stem cell transcription factors

The expression of stem cell transcription factors was detected using two step real time polymerase chain reaction (RT-PCR) analyses. Initially, total RNA was extracted from the sorted cells using a Qiagen AllPrep DNA/RNA Isolation Kit (Qiagen) according to the manufacturer’s instructions. Complementary DNA (cDNA) was synthesized from 1 μg of total RNA using the Transcriptor First Strand cDNA Synthesis Kit (Roche Applied Science, Mannheim, Germany). The random hexamer and anchored-oligo (DT) primers were used. The RT-PCR reaction was prepared using SYBR Green I PCR reagents (KAPA Biosystems, Boston, USA), and the primer for the *Sox2*, *Klf4*, *c-Myc*, *Nanog*, *Oct 3/4*, and GAPDH genes from the Pluripotency Check PCR Primer Set (Clontech Laboratories Inc, Mountain View, USA) were used (Table 1). The RT-PCR reaction was performed using the ABI StepOnePlus™ PCR System (Applied Biosystems, Foster City, USA) under the following procedure: 95 ˚C for 4 min, 40 cycles of 95 ˚C for 15 sec, 60 ˚C for 30 sec, and 72 ˚C for 30 sec. Quantification was performed using the comparative Ct method. The normal stem cell was used as the control sample, and the GAPDH gene was used as the endogenous control.

**Table 1:** **List of primers used in RT-PCR for expression of stem cell transcription factors**

Genes	Forward primer	Reverse primer
**Sox2**	GGTTACCTCTTCCTCCCACTCAG	TCACATGTGCGACAGGGGCAG
**Klf4**	CACCATGGACCCGGGCGTGGCTGCCAGAAA	AAGCTGACTTGCTGGGAACTTGACC
**c-Myc**	CAGAGGAGGAACGAGCTGAAGCGC	TTATGCACCAGAGTTTCGAAGCTGTTCG
**Nanog**	AGGGTCTGCTACTGAGATGCTCTG	CAACCACTGGTTTTTCTGCCACCG
**Oct 3/4**	CTGAGGGCCAGGCAGGAGCACGAG	CTGTAGGGAGGGCTTCGGGCACTT
**GAPDH**	CCGCATCTTCTTGTGCAGTG	CTGTGGTCATGAGCCCTTCC
**Sox2**	GGTTACCTCTTCCTCCCACTCAG	TCACATGTGCGACAGGGGCAG

### In vivo tumourigenicity studies

The ability of the marker-selected cells to initiate *in vivo* tumour development was investigated by subcutaneous transplantation of cells into nude mice. All experiments were carried out using 4–7 week old female NCR nude mice (INVIVOS, Perahu Rd, Singapore). Mice were maintained in individually ventilated cages (IVC) (Allentown Inc., NJ, United States). The experiments were approved by the Universiti Sains Malaysia Animal Ethics Committee according to the institutional guidelines. For the mouse xenograft, 2 × 10^4^ cells from parental cells, putative CSCs, and putative non-CSCs of both A549 and H2170 cell lines were mixed with matrigel (BD Biosciences) and subcutaneously injected into the right flank of the nude mice (n = 3 for each cell type). Mice were monitored every 2 days between two weeks after inoculation. The mice were sacrifice at day 60 or when the tumour diameter reached at least 1 cm in size. All tumour tissues were collected for morphological and histological analysis.

### Microarray analysis

#### Total RNA extraction and cDNA synthesis

Total RNA was extracted from up to 1 × 10^6^ CD166^+^/CD44^+^ and CD166^+^/EpCAM^+^ PHBEC, A549, and H2170 cells using the Qiagen AllPrep DNA/RNA Isolation Kit (Qiagen) according to the manufacturer’s protocol. Briefly, the cells were lysed with lysis buffer and homogenized using the QIAshredder Homogenizer (Qiagen). Ethanol (70%) was then added to the homogenized cell lysates, and the cell lysates were transferred into the RNA spin column. Total RNA that bound to the spin column was eluted from the spin column using RNase free water. The concentration and purity of the extracted RNA were determined using a Nanodrop® ND1000 spectrophotometer, and the RNA integrity number (RIN) was determined using the Bioanalyzer 2100 (Agilent Technologies).

#### ST-cDNA amplification, purification, fragmentation, and labelling

Total RNA (1.5 μg) was amplified using the Applause™ WT-Amp ST System (Nugen Technologies, Inc., San Carlos, USA) following the manufacturer’s protocol. The seven step amplification process produced ST-cDNA, which was further purified using the MinElute Reaction Cleanup Kit (Qiagen). The yield and purity of the purified ST-cDNA were measured using the Nanodrop® ND1000 spectrophotometer. The A260:A280 ratio must be > 1.8 and the concentration must be in the range of 2 to 2.5 μg for the ST-cDNA to be hybridised to the array. The purified ST-cDNA was then fragmented and labelled with biotin (Nugen Technologies).

#### Array hybridisation and scanning

Biotin-labelled fragmented ST-cDNA was hybridised to oligonucleotide probes on Affymetrix GeneChip® 1.0 ST arrays and then washed and stained using the GeneChip® Hybridisation Wash and Stain Kit. For each array, 2–2.5 μg of the fragmented biotin-ST-cDNA were hybridised to the arrays for 17 h at 45°C in a rotating hybridisation oven. The array was stained utilizing the FS450_0007 protocol of the Affymetrix Fluidics Station FS450. The arrays were scanned with an Affymetrix Scanner 3000, and data were obtained using the GeneChip® Operating Software. The microarray experiment was performed using three biological replicates for each sample.

#### Data processing and analysis

Microarray data analysis was performed using GeneSpring GX 7.3.1 software (Agilent Technologies). The CEL file of each array was normalized to the 50th percentile, and probes/genes with expressions less than the 50th percentile were excluded. To identify the significantly regulated genes of putative CSCs, statistical analysis was conducted by comparing the FC of putative CSCs to its normal counterparts (Table 2). The probes/genes then were filtered based on p-value and FC. Probes/genes with p-value < 0.05 and FC > 2.0 were assumed to be significantly regulated. The microarray raw data discussed in this paper were deposited in the NCBI GEO database (Accession number: GSE50627).

**Table 2:** **Comparison groups in microarray data analysis conducted using gene spring software**

Group number	Comparison
1	A549 CD166^+^/ CD44^+^ vs. PHBEC CD166^+^/CD 44^+^
2	A549 CD166^+^ /EpCAM^+^ vs. PHBEC CD166^+^ /EpCAM^+^
4	H2170 CD166^+^ /EpCAM^+^ vs. PHBEC CD166^+^ /EpCAM^+^

### Microarray validation

The differentially expressed genes identified in the microarray analysis were validated by RT-PCR using Taqman® Gene expression assays (Applied Biosystems) in the ABI StepOnePlus™ Real-Time PCR machine. The PCR reactions included 1 μL of 20× Taqman® primer, 10 μL of 2× Taqman® Gene Expression master mix, 2 μL of cDNA template, and 7 μL of RNase free water. The RT-PCR thermal profile was obtained using the following procedure: 50°C for 2 min, 95°C for 20 sec, 40 cycles at 95°C for 15 sec and 60°C for 1 min. Table 3 lists the primer sequences used. The expression level of each target gene in the tested experimental condition (putative lung CSCs) was compared to that of the control condition (PHBEC), and the data was normalized to GAPDH gene expression.

**Table 3:** **List of genes and probe sequences used in the microarray validation assay**

Gene	Primer sequence
CDH2	ATCCTGCTTATCCTTGTGCTGATGT
AKT3	CAAATAAACGCCTTGGTGGAGGACC
EPHX1	GAGGCCTGGAAAGGAAGTTCTCCCT
ITGA3	ACAAGACCACGTGGTTCTCTGTGGA
THBS1	TGAAATACGAATGTAGAGATCCCTA
LAMC2	CTGCATCTGATGGACCAGCCTCTCA
GAPDH	GACTCATGACCACAGTCCATGCCAT

### Functional enrichment analysis

Functional enrichment analysis was performed using DAVID (http://david.abcc.ncifcrf.gov/) [31,32]. Significantly regulated genes (FC > 2; p < 0.05) from each group were submitted to DAVID. The analysis was started by clicking on “Start Analysis” on the header, and the gene list manager panel that appeared was used to perform the analysis step. First, the list of gene IDs was copied and pasted into box A, an appropriate gene identifier type (gene list or background) for the input gene ID was selected, and the submit button was pressed. If DAVID could not recognize more than 20% of the submitted gene ID, the submission was redirected to the DAVID Gene ID Conversion Tool. Once the list of genes was successfully submitted, the analysis of the list was performed using the available DAVID analysis tools. These tools include functional annotation tools, gene functional classification tools, and the gene name batch viewer.

## Results

### Expression of cancer stem cell markers in NSCLC cells

**Figure 1:**
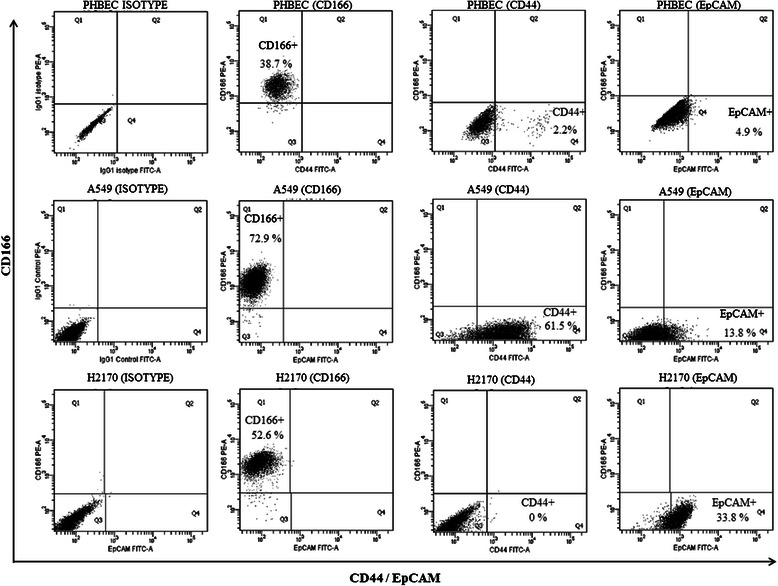
**Identification of CD166**^**+**^**, CD44**^**+**^**, and EpCAM**^**+**^**cells in cancer cell lines (A549 and H2170) and the normal bronchial/tracheal epithelial cell line (PHBEC) by flow cytometry analysis.** A subpopulation of CD166^+^ cells was identified in the PHBEC (38.7%), A549 (72.9%), and H2170 (52.6%) cell lines. A subpopulation of CD44^+^ cells was identified in the PHBEC (2.2%) and A549 (61.5%) cell lines, but CD44^+^ cells were totally absent in the H2170 cell line. In the PHBEC, A549, and H2170 cell lines, 4.9%, 13.8%, and 33.8%, respectively, were EpCAM^+^.

To identify the subpopulation of putative CSCs in cancer cell lines, we investigated the expression of three stem cell surface markers (CD166, CD44, and EpCAM) that previously were described as prominent CSCs markers in lung cancer. Expressions of CD166, CD44, and EpCAM varied among the cell lines. All surface markers except CD44 were expressed in all cell lines, but they exhibited different degrees of expression. CD166 was highly expressed in all cell lines: PHBEC (expressed in 38.7% of cells), A549 (72.9%), and H2170 (52.6%) (Figure 1). CD44 expression was detected only in the PHBEC cells (2.2%) and A549 cells (61.5%) (Figure 1). The expression of EpCAM differed among the cell lines, with the highest expression observed in the H2170 cells (33.8%) followed by the A549 cells (13.8%) and the PHBEC cells (4.9%) (Figure 1).

### Co-expression of CD166 with CD44 and EpCAM to identify the subpopulation of NSCLC cells with stem cell-like properties

**Figure 2:**
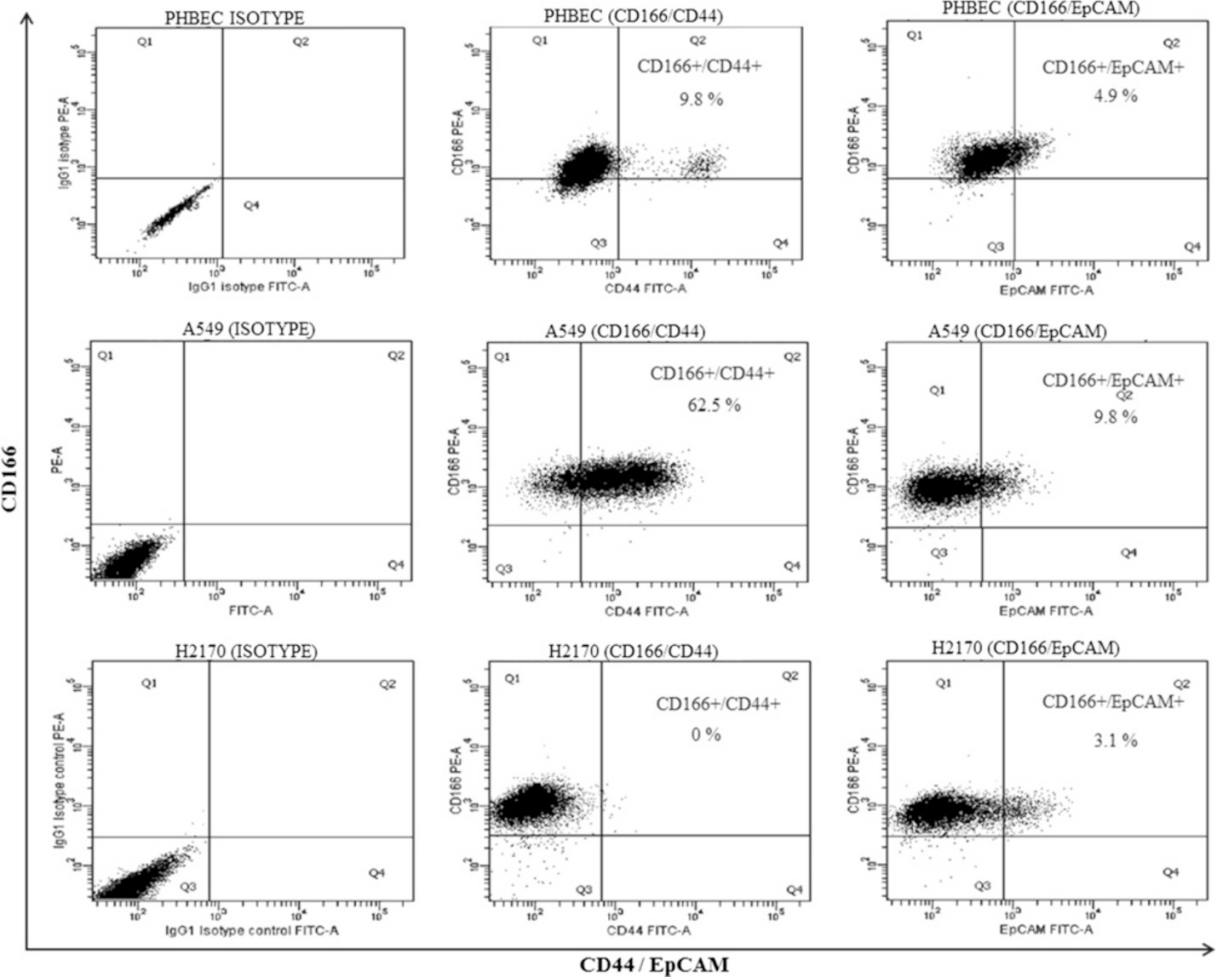
**Flow cytometry analysis of co-expression of CD166/CD44 and C166/EpCAM in the normal bronchial/tracheal epithelial cell line (PHBEC) and cancer cell lines (A549 and H2170).** The cells were stained with anti-CD166 PE, anti-CD44 FITC, and anti-EpCAM FITC.

To identify a more stringent phenotype for the putative CSCs population, co-expression of two markers was investigated. Because the initial analysis using single marker expression showed that CD166 was the prominent marker in both cell lines, we evaluated co-expression of CD166 with CD44 and CD166 with EpCAM. In A549 cells, 62.5% of the cells expressed CD166/CD44 and 9.8% of the cells expressed CD166/EpCAM (Figure 2). In H2170 cells, only expression of CD166/EpCAM was observed in 3.1% of the cells (Figure 2). The double positive cells from the A549 and H2170 cell lines were sorted out and defined as putative lung CSCs. To validate the stemness characteristics of the putative lung CSCs, we also sorted out the double negative population (i.e., CD166^−^/CD44^−^ and CD166^−^/EpCAM^−^) and called this population putative non-CSCs.

### Putative lung CSCs exhibit differentiation potential

**Figure 3:**
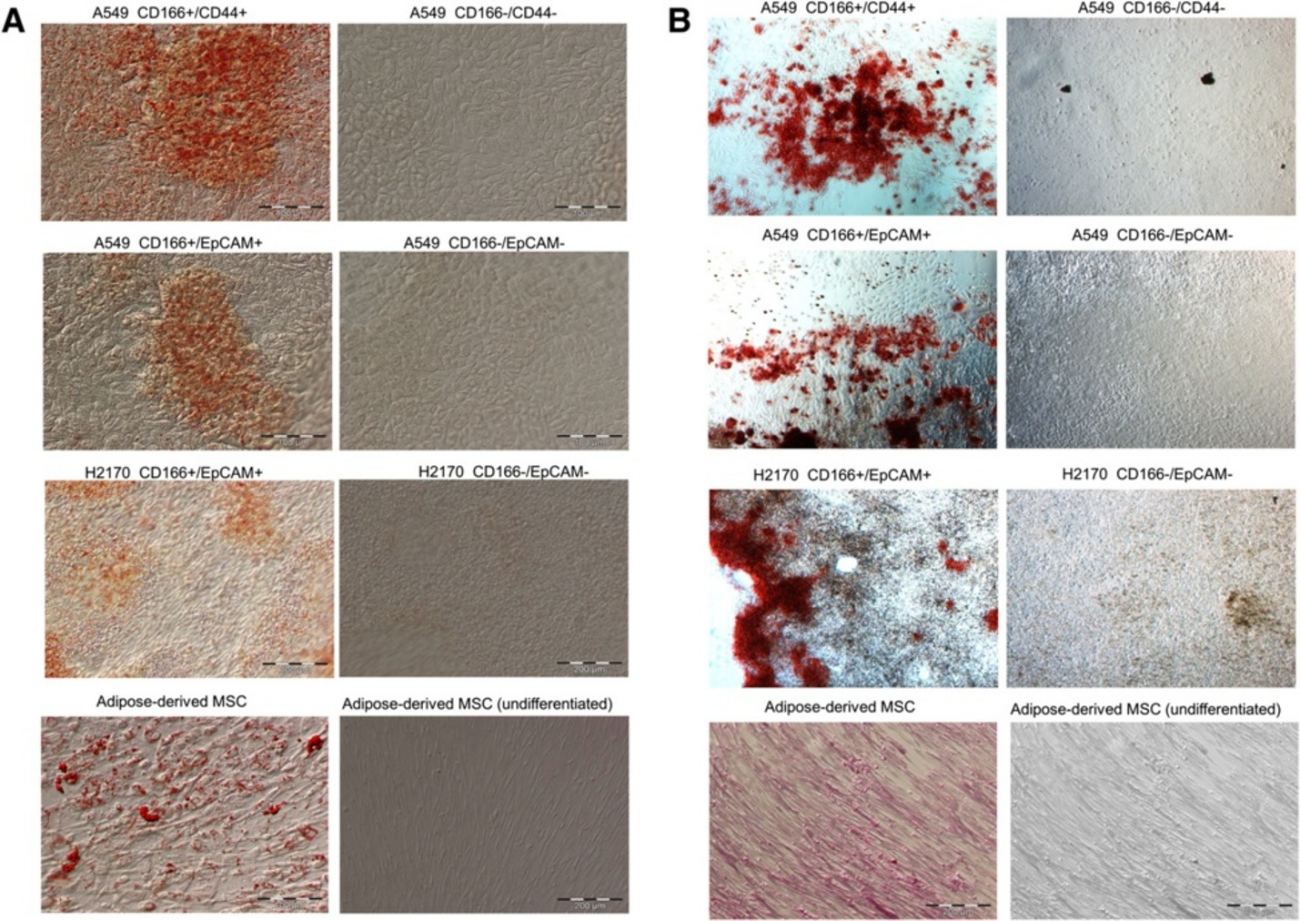
**Adipogenic and osteogenic differentiation potential of putative CSCs from the A549 and H2170 cell lines. (A)** Adipogenic differentiation and **(B)** osteogenic differentiation of putative lung CSCs and putative non-CSCs.

The characteristics of the putative CSCs and putative non-CSCs were assessed by their ability to differentiate into multilineage cells. The cells were induced to differentiate into adipogenic, osteogenic, and chondrogenic cells by culturing them in stem cell differentiation media. Putative CSCs of the A549 and H2170 cell lines were able to differentiate into adipogenic and osteogenic lineages (Figure 3). However, the putative non-CSCs lacked this characteristic (Figure 3). Neither putative CSCs nor putative non-CSCs of both cells lines could differentiate into chondrogenic cells (data are not shown).

### Self-renewal ability of putative lung CSCs

**Figure 4:**
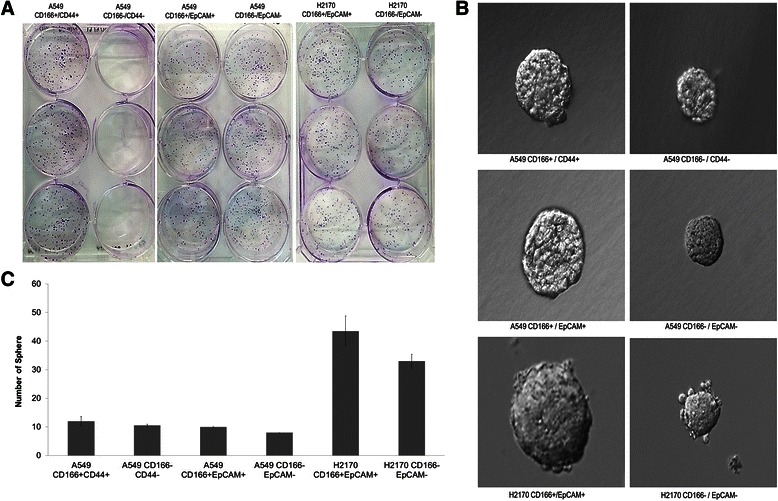
**Self-renewal assay of putative CSCs. (A)** Colony forming assay of putative CSCs. **(B)** and **(C)** show the sphere forming ability of putative CSCs. The error bar indicate the average +/− standard deviation of three independent experiments.

Self-renewal capacity is one of the characteristics of stem cells. The results from the colony formation efficiency assay show that putative CSCs isolated from the A549 and H2170 cells were able to form colonies (Figure 4). However, putative non-CSCs isolated from both cell lines also had the ability to form colonies. We further validated the self-renewal characteristics of the cells by performing the sphere forming assay. After being cultured in serum-free medium supplemented with fibroblast growth factor (bFGF), epidermal growth factor (EGF), and B27 supplement, both putative lung CSCs and putative non-CSCs formed colonies. However, the colony size and the number of colonies formed differed: Putative lung CSCs formed more and larger colonies compared to putative non-CSCs (Figure 4). We concluded that both putative CSCs and non-CSCs have self-renewal ability, but the capability is more prominent and higher in the putative lung CSCs.

### Putative lung CSCs exhibit stem cell gene expression

**Figure 5:**
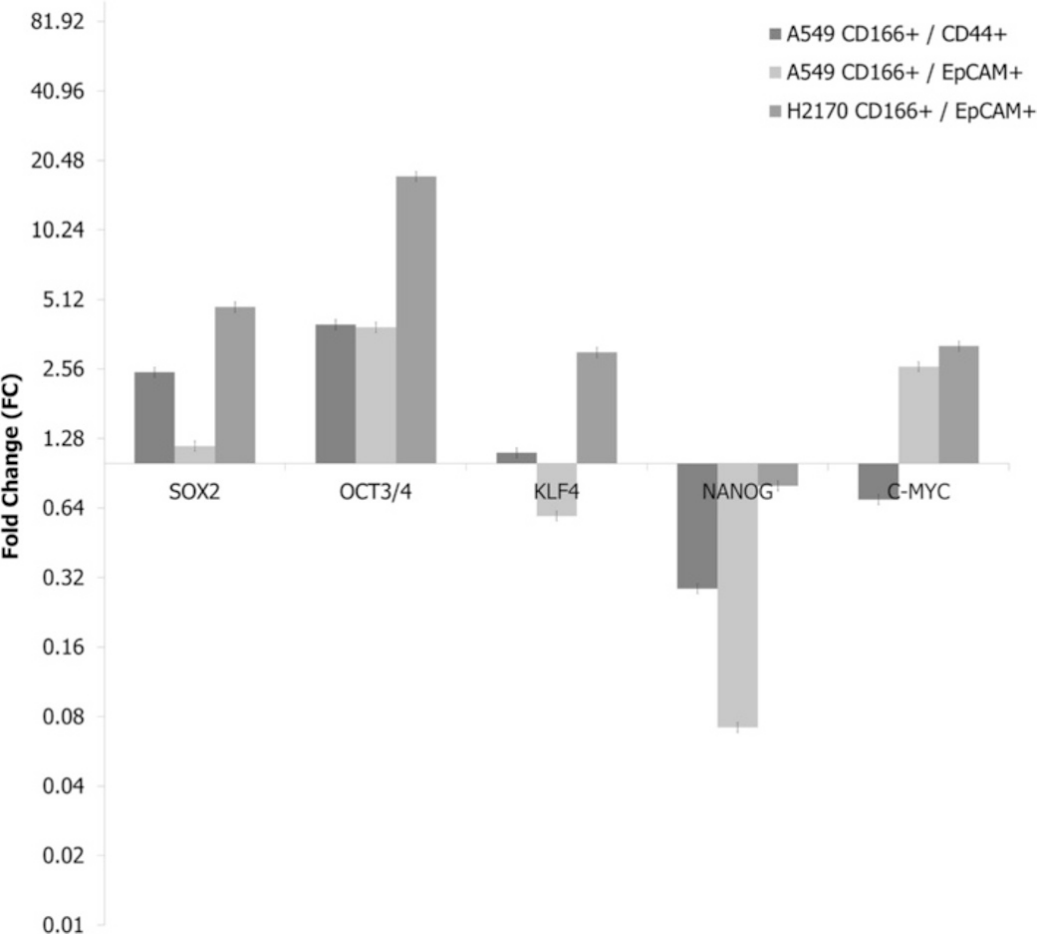
**Analysis of the expression of stem cell related genes in putative CSCs from different cell lines.** Detectable expression levels of the genes were found in all putative CSCs. The PCR reaction without template served as the negative control. The relative expression of target genes was normalized to the level in the normal lung stem cells. The X-axis shows the target genes and the Y-axis shows the fold change. The error bars represent the standard deviation within the triplicate experiments.

The observation that the putative lung CSCs had the ability to differentiate into adipogenic and osteogenic cells led us to investigate whether these cells express stem cell transcription factors such as Sox2, Oct 3/4, Nanog, c-Myc, and Klf4. The expression of the genes was detected using the real-time polymerase chain reaction (RT-PCR) method, and the relative expression of the genes in putative CSCs was compared to the expression of the genes in normal stem cells (PHBEC). Detectable expression levels of these genes were found in putative CSCs of both cell lines (Figure 5). In A549 CD166^+^/CD44^+^ cells, Sox2 and Oct4 were up-regulated with FC values of 2.472 and 3.981 respectively. In A549 CD166^+^/EpCAM^+^ cells, expression of Oct3/4 (3.874) and c-Myc (2.619) was also detected. For H2170 CD166^+^EpCAM^+^ cells, expressions of Sox2 (FC = 4.753), Oct4 (17.484), Klf4 (3.017), and c-Myc (3.213) were up-regulated. The expression of Nanog was down-regulated in all putative CSCs.

### *In vivo* tumourigenicity properties of putative lung CSCs

**Figure 6:**
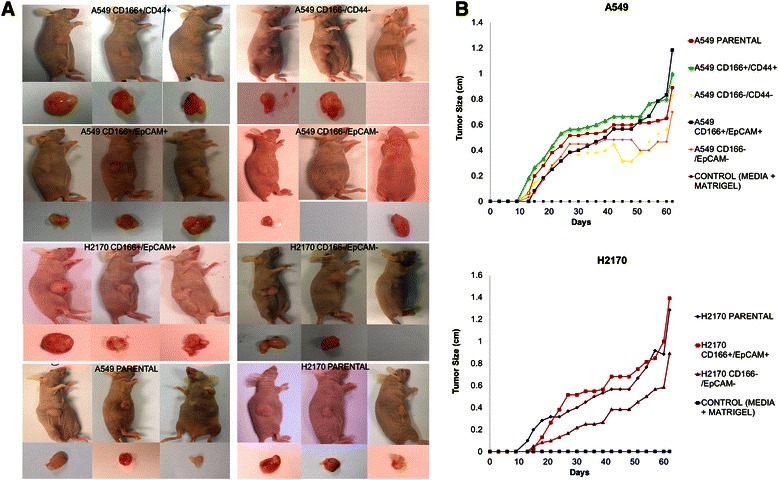
***In vivo*tumourigenecity of putative lung CSCs. (A)** Xenograft tumour resected from the nude mice. **(B)** Graft for the tumour growth experiment. The tumour size was measured using a caliper every two days. The data represent the average value of three animals.

The ability of putative lung CSCs to develop tumours *in vivo* was investigated by subcutaneous transplantation of the cells into nude mice. The injected cells from parental, putative lung CSCs, and putative non-CSCs were able to initiate tumours *in vivo*, but the tumour sizes and tumour incidence differed between the treatments (Figure 6 and Table 4). Putative lung CSCs initiated the growth of larger tumours compared to parental cells and putative non-CSCs. In addition, putative lung CSCs formed tumours in all animals (n = 3), whereas putative non-CSCs formed tumours in two of the three injected animals (Table 4). The tumour growth rate for putative lung CSCs also was higher than those of parental and putative non-CSCs (Figure 6). Therefore, the *in vivo* tumourigenicity experiments demonstrated that the putative lung CSCs were more tumourigenic than the parental and putative non-CSCs.

**Table 4:** ***In vivo*** **tumourigenicity experiment of putative lung CSCs and non-putative CSCs**

Cell type	Cell number injected	Tumour incidence	Tumour size (average) (cm)
A549 Parental	20,000	3 / 3	0.89
A549 CD166^+^/CD44^+^	20,000	3 / 3	1.00
A549 CD166^−^/CD44^−^	20,000	2 / 3	0.83
A549 CD166^+^/EpCAM^+^	20,000	3 / 3	1.18
A549 CD166^−^/EpCAM^−^	20,000	2 / 3	0.70
H2170 Parental	20,000	3 / 3	1.28
H2170 CD166^+^/EpCAM^+^	20,000	3 / 3	1.39
H2170 CD166^−^/EpCAM^−^	20,000	2 / 3	0.89
Control (media + matrigel)	20,000	0 / 3	0

### Transcriptomic profiling of putative lung CSCs using microarray analysis

**Figure 7:**
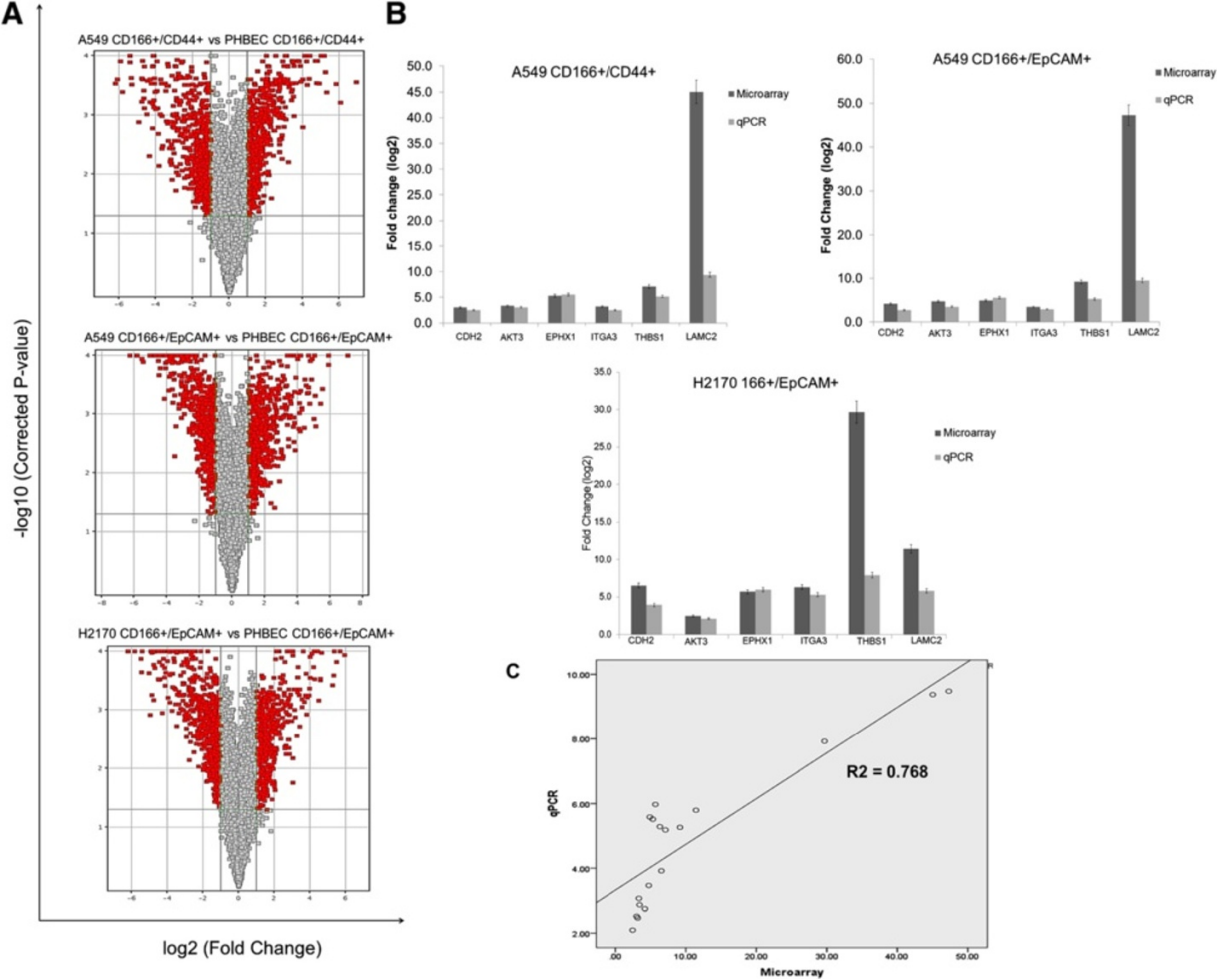
**Microarray analysis and validation. (A)** Volcano plot of differentially expressed genes obtained from microarray analysis. The red coloured dots represent the differentially expressed genes (fold change > 2, p-value < 0.05). **(B)** Validation of the microarray data by RT-PCR for six genes in putative CSCs. Up-regulation of three genes (CDH2, AKT3, and EPHX1) and down-regulation of three genes (ITGA3, THBS1, and LAMC2) were observed in all cells. **(C)** Results of the correlation coefficient test between data from the microarray (log2) and RT-PCR (relative to GAPDH) assays for the genes subjected to the validation study. The X-axis shows the log transformed array data and the Y-axis shows the RT-PCR data for each sample.

The mRNA expression profiles of putative lung CSCs were measured using Affymetrix Expression Console™ software (Affymetrix, Santa Clara, CA, USA), and the data were analysed using GeneSpring software version 12.5 (Agilent Technologies, Santa Clara, CA, USA). The intensity of each array was normalized to the 50th percentile of expression, and the significantly regulated genes were selected using independent *t*-test statistical analysis by comparing the data from the putative CSCs with those from normal lung stem cells. The genes that had a FC > 2.0 and a p-value < 0.05 were considered to be significantly regulated. The lists of significantly regulated genes are shown in the (Additional file 1: Table S1, Additional file 2: Table S2, and Additional file 3: Table S3) for each group. Table 5 summarises the numbers of significantly regulated genes for each putative CSC, and volcano plot analysis was used to visualise the significance and the magnitude of the significantly regulated genes (Figure 7). The number of significantly regulated genes ranged from 1229 to 1335, and the number of down-regulated genes was higher than that of up-regulated genes.

**Table 5:** **Number of significantly regulated genes (p < 0.05, fold change > 2.0) in putative CSCs compared to putative normal stem cells**

Groups	Number of genes		
	Total	Up-regulated	Down-regulated
A549 CD166^+^/CD44^+^ vs. PHBEC CD166^+^/CD44^+^	1229	598 (48.65%)	631 (51.34%)
A549 CD166^+^/EpCAM^+^ vs. PHBEC CD166^+^/EpCAM^+^	1335	677 (50.71%)	658 (49.28%)
H2170 CD166^+^/ EpCAM^+^ vs. PHBEC CD166^+^/ EpCAM^+^	1292	547 (42.33%)	745 (57.66%)
**Total**	**3856**	**1822**	**2034**

### Validation of microarray data by RT-PCR

To verify the expression value of the microarray data, the original amplified RNA samples used for microarray analysis were validated for six genes using RT-PCR. Three of the selected genes were up-regulated and three were down-regulated. The expression values detected by both microarray and RT-PCR techniques were plotted as log2 FC (Figure 7). The Pearson correlation coefficient test showed that the expression values detected by both platforms were in agreement (p < 0.05) (Figure 7).

### Functional enrichment analysis of differentially expressed genes in putative CSCs

**Figure 8:**
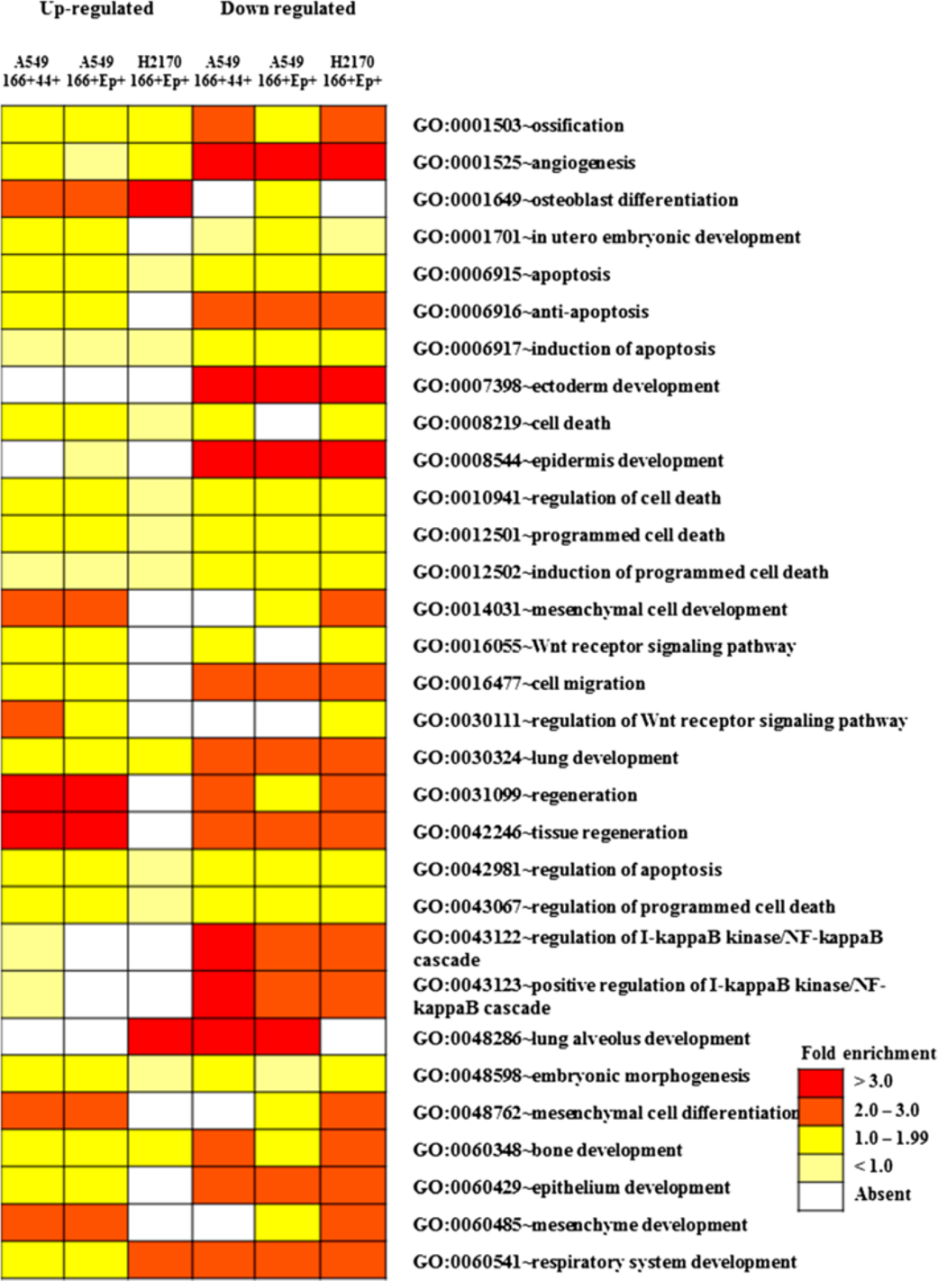
**Gene ontology (GO) terms for the biological function of significantly regulated genes from putative CSCs.** The level of significance is represented by the value of fold change.

To gain a better understanding of the functions of significantly regulated genes in lung CSCs, we conducted bioinformatics analysis using the Database for Annotation, Visualization, and Integrated Discovery (DAVID) programme. We looked at the gene ontology (GO) terms for biological function and the Kyoto Encyclopaedia of Genes and Genomes (KEGG) pathways that are associated with the CSCs gene list. Up- and down-regulated genes in all three putative CSCs (A549 CD166^+^/CD44^+^, A549 CD166^+^/EpCAM^+^, and H2170 CD166^+^/EpCAM^+^) were found to be involved in several biological cancer processes, including angiogenesis, apoptosis, anti-apoptosis, induction of apoptosis, cell death, and cell migration. In addition, the three putative CSCs were found to share several development and stem cell related biological processes, such as ectoderm development, epidermis development, osteoblast differentiation, mesenchymal cell development, Wnt receptor signaling, lung development, regulation of the NF-kappa β cascade, and bone development (Figure 8).

A more informative analysis of functional annotation was achieved by studying the enrichment of differentially expressed genes in a particular pathway. The up-regulated genes were involved in cancer, ATP-binding cassette (ABC) transporter, Wnt signaling, drug metabolism, and NSCLC pathways (Table 6). The significant pathways for the down-regulated genes were the p53 signaling, apoptosis, Hedgehog signaling, ECM-receptor interaction, cancer, and SCLC pathways (Table 6).

**Table 6:** **Common significant pathways associated with up-regulated and down-regulated genes of putative CSCs**

Pathway ID	Pathways	Fold enrichment		
		A549 CD166^+^/CD44^+^	A549 CD166^+^/EpCAM^+^	H2170 CD166^+^/EpCAM^+^
*Up-regulated*				
hsa00982	Drug metabolism	3.661	–	–
hsa02010	ABC transporters	3.041	–	–
hsa04010	MAPK signalling pathway	–	1.933	1.870
hsa04310	Wnt signalling pathway	1.063	1.025	-
hsa04514	Cell adhesion molecules (CAMs)	1.419	–	1.719
hsa04520	Adherens junction	1.390	–	–
hsa05200	Pathways in cancer	–	0.944	1.384
hsa05223	Non-small cell lung cancer	–	1.912	4.203
*Down-regulated*				
hsa04115	p53 signalling pathway	3.935	2.589	2.660
hsa04210	Apoptosis	1.957	2.783	2.079
hsa04340	Hedgehog signalling pathway	1.737	–	–
hsa04512	ECM-receptor interaction	3.765	3.668	3.589
hsa05200	Pathways in cancer	2.225	1.946	1.960
hsa05222	Small cell lung cancer	2.027	2.096	2.392

## Discussion

Because CSCs likely play an important role in maintaining cancer cell populations, targeting specific components of CSCs regulatory pathways could open up a new strategy for cancer treatment. Identification and isolation of CSCs from NSCLC cells is the initial step in identifying more specific CSCs markers in the NSCLC cell population. We found that CD166 was highly expressed in both normal and lung cancer cells, and we used the combinations of CD166/CD44 and CD166/EpCAM for further analysis.

The flow cytometry analysis revealed that the lung cancer cells consist of a heterogeneous population with different phenotypes (CD166^+^/CD44^+^, CD166^+^/CD44^−^, CD166^−^/CD44^+^, CD166^−^/CD44^−^, CD166^+^/EpCAM^+^, CD166^+^/EpCAM^−^, CD166^−^/EpCAM^+^, and CD166^−^/EpCAM^−^), which supports the initial hypothesis that the CSCs population is heterogeneous. We hypothesized that the double positive cells (i.e., CD166^+^/CD44^+^ and CD166^+^/EpCAM^+^) are the putative lung CSCs in A549 and H2170 cells. The putative lung CSCs showed multilineage differentiation and self-renewal capability, which proved that they had the stem cell-like phenotype.

Results of previous studies support the use of CD166, CD44, and EpCAM as CSCs markers. The combination of CD166/CD44 was previously used to identify CSCs from colorectal cancer cell lines; CD166^+^/CD44^+^ cells were found to have higher clonogenicity and accelerated tumour development compared to CD166^−^/CD44^−^ cells, and the observation was cell dependent [33]. To date, there have been no reports of the combination of CD166/CD44 and CD166/EpCAM to identify lung CSCs, but co-expression of other CSCs markers to identify lung CSCs has been reported. For instance, Wang et al. combined CD44 and CD90 to identify lung CSCs [22]. They demonstrated that CD44^+^/CD90^+^ cells had therapy resistance and higher colony and spheroid forming potential when compared to CD44^+^/CD90^−^, CD44^−^/CD90^+^, and CD44^−^/CD90^−^ cells. Another study combined CD133 with CD44 to identify lung CSCs in A549 cells and found that CD133^+^/CD44^+^ cells had significant CSCs properties (i.e., continuous proliferative capacity and differential potential) [34].

In this study, the expression of surface markers differed between the tested cell lines, even though both are NSCLC cell lines. For example, 61.5% and 0.0% of A549 and H2170 cells, respectively, were CD44^+^. Other studies using the same A549 cells reported that 84.41% [35] and 0.0% [15] of A549 cells expressed CD44. Stuelten et al. also found inconsistent expression of CD44 in nine NSCLC cell lines, including A549 cells [35]. In addition, different expression levels of CD44 have been reported in other types of tumours, including colon, ovarian, and breast cancers [35]. We also found that CD166 and EpCAM expression differed between cell lines. We detected expression of CD166 in 72.9% and 52.56% of A549 and H2170 cells, respectively, whereas the values for EpCAM were 13.8% and 33.8%, respectively. The inconsistent expression profiles of CSCs markers among different studies could be related to individual cancer variations, different potency states, and functional characteristics of the CSCs population. Thus, in the absence of a specific marker, the true percentage of CSCs in a tumour, particularly in long-established cancer cell lines, is controversial [36]. The variation in environmental and selective pressures experienced by cancer cells *in vitro* and *in vivo* might trigger or suppress different pathways that regulate CSCs functions, and it is not clear whether the CSCs profiles could vary with circumstances.

The abilities of the CD166^+^/CD44^+^ and CD166^+^/EpCAM^+^ A549 and H2170 cells to differentiate into adipogenic and osteogenic lineages and to express stem cell transcription factors showed their stem cell characteristics. Moreover, the increased tumorigenic ability of the putative lung CSCs as shown in *in vivo* study also indicated their cancer stem cells characteristic. Microarray analysis of putative lung CSCs was carried out to better understand the transcriptomic regulation of putative CSCs as compared to normal lung stem cells. Using microarray technology, transcription profiling was performed for putative lung CSCs isolated from the two lung cancer cell lines. The composition of the up-regulated genes and their pathways showed that these genes play an important role in maintaining the stemness of the lung CSCs, whereas the dysregulated genes were involved in cellular repair. The GO term analysis showed consistent results, with the significantly regulated genes of putative CSCs being involved in stem cell related biological and development processes. These findings clearly illustrate that CSCs are present in lung cancer cells and that these isolated putative CSCs possess stem cell characteristics.

The significantly regulated genes identified in our study were involved in the Wnt and hedgehog pathways and were associated with the self-renewal process of normal stem cells [37]. The ability to self-renew is a special characteristic of stem cells, as it allows them to divide and maintain their stemness. Wnt and hedgehog signaling pathways have been reported to play an important role in carcinogenesis as well as in normal stem cell processes [4,38]. In addition, the over-expression of Wnt signaling molecules has been reported to be involved in drug resistance of A549 cells, and up-regulation of this signaling pathway was suggested to be one of the characteristics of CSCs [39]. Our microarray data showed that Wnt signaling was up-regulated only in putative CSCs of A549 cells; it was not detected in H2170 cells. This could explain the higher colony forming ability of A549 cells compared to H2170 cells.

The ECM interaction pathway is another pathway that showed a strong relationship with the putative CSCs transcriptome. Our data showed that this pathway was consistently conserved in putative CSCs regardless of cell type. The ECM is a non-cellular component of cells that consists of a complex mixture of structural and functional macromolecules, including proteins, glycoproteins, proteoglycans, and polysaccharides. The ECM plays an important role in tissue and organ morphogenesis and in the maintenance of cell and tissue structure and function [40,41]. In the present study, the genes involved in the ECM pathway, including laminin and integrins, were down-regulated (Table 7). It also has been suggested that the ECM is a non-cellular component of the adult stem cell niche [42,43]. The ECM plays a role in maintaining stem cell properties and in regulating stem cell differentiation [42,43]. The dysregulation of the ECM, which causes an imbalance between stem cell self-renewal and differentiation, might lead to the formation of CSCs. In addition, the dysregulation of ECM was associated with development and progression of tumours, as it promotes the tumour microenvironment [44]. An abnormal ECM also indirectly affects cancer cells by influencing the behavior of stromal cells, endothelial cells, immune cells, and fibroblasts, which are the main initial culprits that cause abnormal ECM production [44-46].

**Table 7:** **Genes involved in the ECM pathway**

Gene symbol	Gene name	Fold change		
		A549 CD166^+^/CD44^+^	A549 CD166^+^/EpCAM^+^	H2170 CD166^+^/EpCAM^+^
ITGA2	Integrin, alpha 2	−6.453101	−6.003461	−5.65315
ITGA3	Integrin, alpha 3	−3.122479	−3.464945	−6.222598
ITGA5	Integrin, alpha 5	−3.526326	−3.174653	−5.131281
ITGA6	Integrin, alpha 6	−3.517541	−3.317876	−43.17514
ITGB4	Integrin, beta 4	−6.819779	−5.698617	−6.173566
ITGB6	Integrin, beta 6	−22.19036	−30.26098	−3.902746
ITGB8	Integrin, beta 8	−5.458705	−4.551452	−5.533338
LAMA3	Laminin, alpha 3	−4.648527	−4.327131	−2.236711
LAMB3	Laminin, beta 3	−8.490638	−8.384007	−4.292205
LAMC2	Laminin, gamma 2	−42.03006	−46.44047	−11.52965
TNC	Tenascin C	−8.586228	−7.472796	−6.891974
THBS1	Thrombospondin 1	−6.843357	−8.93955	−29.22261

## Conclusions

We successfully identified and characterised putative CSCs from NSCLC cells. The CSCs with the CD166^+^/CD44^+^ and CD166^+^/EpCAM^+^ phenotypes have the ability to differentiate into adipogenic and osteogenic lineages and possess self-renewal ability. The gene expression study revealed that the putative lung CSC transcriptome is significantly associated with stem cells and cancer biological processes, including angiogenesis, migration, pro-apoptosis and anti-apoptosis, osteoblast differentiation, mesenchymal cell differentiation, and mesenchyme development, and is involved in stem cell self-renewal pathways, Wnt and hedgehog signaling, and cancer-associated pathways. This study revealed that isolated putative lung CSCs exhibit the characteristics of multipotent stem cells, and their genetic composition might be valuable for future gene and stem cell therapy for lung cancer.

## Additional files

Additional file 1: Table S1. List of significantly regulated genes of A549 CD166+/CD44+.
Additional file 2: Table S2. List of significantly regulated genes of A549 CD166+/EpCAM+.
Additional file 3: Table S3. List of significantly regulated genes of H2170 CD166+/EpCAM+.
